# Evaluating social network metrics as indicators of tail injury caused by tail biting in growing-finishing pigs (*Sus scrofa domesticus*)

**DOI:** 10.3389/fvets.2024.1441813

**Published:** 2024-09-27

**Authors:** Kaitlyn M. St. Charles, Kimberly L. VanderWaal, Jon E. Anderson, Lee J. Johnston, Yuzhi Z. Li

**Affiliations:** ^1^Department of Animal Science, College of Food, Agriculture, and Natural Resource Sciences, University of Minnesota-Twin Cities, St. Paul, MN, United States; ^2^Department of Veterinary and Biomedical Sciences, College of Veterinary Medicine, University of Minnesota-Twin Cities, St. Paul, MN, United States; ^3^Department of Veterinary Population Medicine, College of Veterinary Medicine, University of Minnesota-Twin Cities, St. Paul, MN, United States; ^4^University of Minnesota-Morris, Morris, MN, United States; ^5^West Central Research and Outreach Center, College of Food, Agriculture, and Natural Resource Sciences, University of Minnesota, Morris, MN, United States

**Keywords:** pig, behavior, tail biting, social structure, animal welfare

## Abstract

Tail biting is a multifactorial behavior that causes welfare and economic challenges in swine production. As of 2024, research exploring the influence of pig social structure on the development of tail biting is limited. The objective of this study was to explore whether social structures of pigs from different litter origins can impact tail biting and, ultimately, tail damage. Pigs (*n* = 96) were grouped (eight pigs/pen) based on their litter origin: non-littermates (NLM), half-littermates, and littermates (LM). Tail injury scores were assessed twice weekly from 10 to 24 weeks of age, with a maximal tail injury score (MTS) over the study period being used to evaluate victimization by tail biting. Pig behavior was video-recorded at 15, 19, and 23 weeks of age. Association networks based on lying behavior and tail biting interaction networks were evaluated at pen-and pig-levels using social network analysis. Pigs in LM pens experienced higher median MTS compared to pigs in NLM pens (Median = 1.5; Interquartile range = 1–2; *p* = 0.009). Within association networks, NLM pens had lower degree centralization measures than other pens at both 15 (Estimated marginal mean [EMM] = 0.07; 95% CI = 0.02–0.12; *p* = 0.003) and 23 weeks (EMM = 0.09; 95% CI = 0.04–0.14; *p* = 0.01) and pigs in NLM pens had higher weighted degree centrality than those in other pens (EMM = 1.00; 95% CI = 0.90–1.11; *p* = 0.002), suggesting pigs in NLM pens had more uniform, stronger, and more connections with their pen-mates. In tail biting networks, increased weighted in-degree centrality was associated with increased odds of pigs receiving a more severe MTS (OR = 1.56; 95% CI = 1.08–2.27; *p* = 0.02). Pigs with increased weighted out-degree centrality tended to have increased odds of receiving a more severe MTS (OR = 1.19; 95% CI = 0.97–1.48; *p* = 0.09). These preliminary data suggest a potential relationship between social structures and tail biting in growing-finishing pigs.

## 1 Introduction

Pigs are social animals that form social structures to maintain group stability (1). After the initial establishment of dominance hierarchies through agonistic interactions, maintenance of social structures in pigs is facilitated mainly by threats and avoidance (2). To a lesser extent, pro-social behaviors, such as affiliative behaviors [i.e., peaceful or friendly behaviors shared between animals (3) (e.g., social nosing in pigs) (4)], are hypothesized to play a role in maintaining social structures in animals (4, 5). However, this hypothesis has only been explored in pigs in a limited capacity (6). In previous work, formation of affiliative associations among pigs has been assessed based on spatial proximity, from which researchers have suggested that pigs can form and loosely maintain preferential associations within a group (7, 8). Additionally, preferential associations among pigs can vary based on sex, age, position of the pig in a dominance hierarchy, and the behavioral context (6, 9). Further, impacts of relatedness of pigs on formation and maintenance of preferential associations have been explored but are not conclusive (8, 10). Also of consideration is that in previous work, the effect of litter origin on preferential association has often been confounded with the effect of immediately mixing unfamiliar pigs (8, 10). Consequently, one cannot clearly delineate when effects of litter origin or effects of mixing are impacting preferential associations formed among pigs. Moreover, there has been limited research conducted that has assessed the impact of litter origin on social structure well beyond the time when unfamiliar pigs have been mixed.

As researchers seek to better understand the influences of social structure in pigs, more novel tools to explore livestock social structures are being employed such as the use of social network analysis. Study of pigs’ social structures using social network analysis began in earnest in the 2010’s and continues to increase in animal welfare science today (11–13). Swine behaviorists have utilized social network analysis (SNA) to analyze agonistic behavior networks that were observed during the post-mixing period when pigs are actively establishing a social hierarchy (14–19). However, other network types have also been explored such as play fighting networks (20), association networks (8), and tail biting interaction networks (21). While dyadic-level analyses have also been employed to assess social preferences beyond the post-mixing period when social structures are more stable (6), social network analyses at pig and pen-levels are still underutilized in assessing pigs’ social structures and preferences during periods of social stability. Such exploration is important because there can be variability in the stability of established social structures due to various factors (e.g., age and timing of piglet socialization) (22, 23). Additionally, unbalanced social structures may lead to spikes in agonistic behaviors (24) and potentially induce abnormal behaviors such as tail-biting (16, 25).

Tail biting behavior is commonly observed in growing-finishing pigs, and creates significant welfare and health consequences if no interventions occur (26, 27). Tail biting behavior and outbreaks of tail biting have been reduced by providing pigs with appropriate environmental enrichment (28); however, tail biting behavior is widely acknowledged as being influenced by multiple internal and external factors (e.g., sex as an internal factor and feeding frequency as an external factor) (28–30). Tail docking still prevails in North America and some other major pig-producing regions of the world as a method to prevent pigs from tail biting in conventional swine production systems. Tail docking is often the most convenient intervention because farms that use liquid manure handling systems cannot provide straw bedding, an effective environmental enrichment used to combat tail biting. Tail docking is painful to piglets and is considered an animal welfare concern; thus, alternative strategies to tail docking that do not exclusively rely on provision of environmental enrichment are needed (31).

Similar to how dyadic agonistic interaction networks of pigs can be used to predict skin lesions in specific contexts (20), tail injury caused by tail biting might also be predicted using social network metrics. The relationship between affiliative behaviors and tail biting behavior in pigs is not well understood, but such connections have been hypothesized (8). Piglets involved in tail biting behavior are more engaged in social interactions (e.g., pig-to-pig contacts) with pen-mates than piglets not as involved in tail biting behavior (32), which suggests a connection between tail biting and social behavior.

However, the impact of proximity of pen-mates on tail biting behavior is not clearly understood, with proximity potentially being beneficial or detrimental as it relates to the emergence of tail biting behavior. Taylor et al. (29) theorized that tail biting is linked to misplaced foraging behavior. Thus, close proximity between pigs could result in tail biting between such pigs if a neighbor’s tail is a readily accessible object to direct misplaced foraging behaviors toward. However, pro-social behaviors based on spatial proximity may help pigs cope with stress (33); so, increased association with other pigs could potentially aid in alleviating stress-induced abnormal behaviors, such as tail biting. As is observed for other pig behaviors, such as nosing (4), conclusive connections between tail biting behavior and social structures in growing-finishing pigs have yet to be elucidated.

The ultimate goal of this study is to explore whether tail injury from tail biting can be indicated using social network analyses, specifically analyses of two behavioral networks: association networks and tail biting interaction networks. Given the potential impact of litter origin on affiliative associations among pigs (8), the first objective of this study was to assess if maintaining litter origin groups is associated with tail injuries caused by tail biting and might impact association networks or tail biting interaction networks. The second objective was to explore whether tail injury scores and/or if pigs becoming a victim of tail biting could be indicated by social network metrics. In this exploratory study, we hypothesize that litter origin influences social preferences and tail biting interactions among growing-finishing pigs and that social network metrics have the potential to serve as indicators of tail biting.

## 2 Materials and methods

The Institutional Animal Care and Use Committee of the University of Minnesota reviewed and approved the experimental protocol of this study (IACUC# 1602-33462A). The study was conducted at the University of Minnesota’s West Central Research and Outreach Center (WCROC) located in Morris, MN. All animals involved in the study were born at the WCROC.

### 2.1 Animals, housing, and management

Pigs (*n* = 96; 12 pens of eight pigs total; Landrace × Yorkshire × Duroc) from 30 litters born in a confinement farrowing barn were used in the present study. Piglets’ tails were left intact. At 4 weeks of age, piglets were weaned and transferred to a confinement nursery barn. Housing and management specifications within the farrowing barn and the nursery barn are as previously described in Li et al. (8). At 10 weeks of age, pigs were moved from the nursery barn to an intensively managed growing-finishing barn. In the growing-finishing barn, each pen housed eight pigs and was equipped with a dry feeder with four access spaces, a nipple drinker, and fully slatted concrete floors. Floor space allowance was 0.86 m^2^/pig excluding space occupied by the feeder.

In the growing-finishing barn, pigs were provided free access to corn-soybean meal-based diets that were formulated to meet or exceed nutritional requirements of pigs recommended by the National Research Council (34). Room temperature was maintained within the thermoneutral zone for pigs as much as possible with mechanical ventilation and heating systems. Average daily room temperature was 26 C (SD = 1.6 C, range from 22 to 30 C) during the study period. When room temperature reached 30.5 C, sprinklers on the ceiling were triggered to drip water and help pigs dissipate heat through evaporation. Light period was 8 h daily starting at 0800 h. Pigs were not provided with environmental enrichment. General health of pigs (as indicated by activities, blood on the tail or any other injury) and function of feeders and drinkers were checked twice daily in the morning and afternoon by farm animal attendants. When any pig in a pen was observed with blood on the tail, a trained researcher assessed tail injury of all pigs in the pen using the scoring system described in Table 1. Pigs remained in the growing-finishing barn until they reached market weight at 24 weeks of age.

**Table 1:** Tail injury scoring system^1^ and associated descriptions of tail condition for each score.

Tail injury score	Tail injury description
0	No observable damage or injury
1	Healed lesions, minor scabs, or scars
2	Evidence of chewing and/or puncturing; Observable blood but no signs of infection
3	Evidence of chewing and/or puncturing; Observable blood with visual signs of infection
4	Complete or partial loss of tail with visual signs of infection

1 Adapted from Kritas and Morrison (35) and Li et al. (8).

### 2.2 Experimental treatment and design

At weaning, pigs without tail injury were assigned to three treatments based on litter origin: (1) Littermates (LM), (2) Half-littermates (HLM), and (3) Non-littermates (NLM), as described previously by Li et al. (8). Littermate pens consisted of 8 pigs that were farrowed and nursed by the same sow. Half-littermate pens consisted of two sets of four pigs (two barrows and two gilts per set) farrowed and nursed by one sow per set. Non-littermate pens consisted of eight pigs that were each farrowed and nursed by a different sow. Eight to nine pens of each treatment, with four barrows and four gilts per pen were formed at weaning into the nursery barn. Pigs were housed within their litter origin treatments throughout nursery and growing-finishing periods.

Upon moving to the growing-finishing barn, treatment groups were assigned randomly to camera-equipped pens using a random number generator. Data were collected from four pens per litter origin treatment that did not have any pigs with tail damage when entering the growing-finishing phase. Pigs were not mixed in the growing-finishing barn. Pigs were housed with their pen-mates from weaning until they reached market weight.

### 2.3 Data collection

#### 2.3.1 Growth performance, tail injury score, and victimization by tail biting

The study period for data collection started when pigs were transferred to the growing-finishing barn at 10 weeks of age and ended when pigs reached market weight at 24 weeks of age. Pigs were weighed individually at 10 weeks of age, every 4 weeks thereafter, and at the conclusion of the study, from which average daily gain of individual pigs was calculated. Feed consumed on a pen-basis was recorded over the same period as body weight and average daily feed intake was calculated on a pen basis. Gain efficiency (Gain:Feed) was calculated based on average daily gain and average daily feed intake on a pen-basis.

To evaluate tail damage caused by tail biting behavior, tail injury was scored using a subjective system described by Kritas and Morrison (35) and Li et al. (8). The scoring system used a scale ranging from 0 to 4, with injury scores describing an increasing severity of injury as the scale progresses from 0 to 4 (Table 1).

All pigs were assessed for tail injury when they were transferred to the growing-finishing barn, and twice weekly (Monday and Friday) thereafter until the end of the study period by a trained researcher. In the event that any pig received a tail score of 2 or higher, all pigs in that pen were scored daily for progress of tail damage until the tail with a score of 2 or higher was healed or the pig was removed from the study due to tail infection. The greatest tail injury score that each pig received at any time point in the growing-finishing barn was noted and defined as the maximal tail score (MTS) for that pig. The MTS was also used to categorize victimization status of tail bitten pigs during the study. Pigs that received a MTS of two or greater were considered a victim of tail biting (8).

#### 2.3.2 Behavior

Pig behaviors were captured during the finishing phase (i.e., 15 weeks until processing) via video recordings using Infrared Color Bullet Cameras (TruVision High Definition Camera—1080p Bullet, United Technologies, Farmington, CT, United States) which were attached to the back wall of each pen and angled down to capture the entire area of the pen. Cameras were connected to a computer equipped with GeoVision recording software (GeoVision Multicam Digital Surveillance System V8.2; United States Vision Systems Inc., Irvine, CA, United States) that recorded 12 frames/s.

Pigs were allowed 4 weeks to adapt to the new environment of the growing-finishing barn prior to video recording. Pig behavior in each pen was recorded for three 6-h periods (from 0900 to 1500 h) during the study at four-week intervals starting at 15 weeks of age, with the remaining video-recording periods occurring at 19, and 23 weeks of age. Before each video recording period, pigs were marked on their backs with unique shapes using non-toxic, animal-safe paint to allow clear identification of individual pigs during filming. Routine animal management tasks were performed (from 0700 to 0800 h) before video recording started so that pigs were not interrupted during video recording. One week after each video-recording day, pigs within a pen were moved to an identical pen within the same room to control for potential spatial preferences unrelated to social association (8).

Video-recordings were viewed by two trained researchers, with one researcher registering lying posture and whether any pigs were lying together, and the other researcher registering tail biting events. Researchers were blind to treatment groups when viewing video recordings to avoid subjective bias. To register lying posture of individual pigs and pairs of pigs lying together, the researcher scanned all video-recordings at 10 min intervals using the instantaneous scan sampling method as described by Martin and Bateson (36). Individual pigs in the pen were identified and registered for lying behavior (yes = 1; no = 0) and for lying together with another identified pig (yes = 1; no = 0) in a square binary matrix. Lying behavior was defined as a pig being in a state of lateral or ventral recumbency and two pigs were considered lying together if they were lying parallel/inverse parallel with more than 50% of their bodies touching each other (8).

Thirty-six matrices (6 scans/h × 6 h) were obtained for each pen on each observation day. These matrices were aggregated into one matrix to represent the 6-h observation period. Three aggregated matrices of lying together data were constructed per pen (one for each observation period over the course of three weeks). Thus, 36 aggregated matrices of lying data together across 12 pens were summarized for these observation days. Lying posture data were used to calculate a lying time budget. Time budget of lying was estimated by the number of times that a pig was observed lying as percent of total number of scans over the 6 h during each recording day.

A researcher examined all video recordings continuously to capture tail biting events. A tail biting event was defined as an instance in which a pig (initiator of a tail biting event) had the tail of another pig (recipient of a tail biting event) in its mouth and was biting or pulling hard enough to cause a reaction in the recipient pig (37). When a tail biting event occurred, the biter and the recipient were identified and recorded into a matrix. All tail biting events that occurred in a pen during each 6-h video recording were recorded on the same matrix for each observation period. Thirty-six matrices of tail biting interactions across 12 pens were summarized over the three observation periods conducted.

The initial 36 matrices of tail biting interactions were used for network matrix construction, with one matrix constructed per pen per recording period. Due to the low incidence of tail biting events observed, tail biting matrices were aggregated over the three observation periods to avoid completely sparse matrices. Consequently, one matrix of tail biting interactions was constructed per pen over the entire study period, resulting in 12 network matrices of tail biting interactions for 12 pens.

#### 2.3.3 Network matrix construction

Two types of network matrices were constructed: association networks and tail biting interaction networks. Associations among pigs were defined by spatial proximity to other pigs when lying (7, 8). To determine associations among pigs in a pen, a half weight association index (HWI) value was calculated from the lying together data and time budget using Equation 1 (7, 8) where *x* is the number of times pig *a* and pig *b* are lying together during 6 h of observation, *n_a_* is the number of times pig *a* is lying during the same period, and *n_b_* is the number of times pig *b* is lying during the same period.


(1) $$HWI=\frac{x}{\left(\frac{n_{a}+n_{b}}{2}\right)}$$

Half weight association index values were calculated in Microsoft Excel (version 16.66.1) for each pig in each pen. All matrices of raw data built around frequency of lying together were transformed to 36 matrices of HWI values. These values were used to provide weight (i.e., strength of the connection between individuals, see Section 2.3.4 Social Network Analysis Metric Calculation below) within association networks.

#### 2.3.4 Social network metric calculation

Social network analysis was used to calculate pen- and pig-level metrics within the association and tail biting interaction networks. Social network analysis is a methodology rooted in graph theory and is used commonly in numerous fields of study including social sciences (38), epidemiology (39), and ecology (40). This methodology has been used previously to understand behavioral patterns in swine systems (14, 18, 20, 21). At a basic level, SNA explores how different nodes (i.e., individuals) within a group connect to each other via edges (i.e., relations or interactions), with these connections ultimately forming a network system (41). Edges may have directionality such that a connection may start at one node and end at another node. For example, a behavioral interaction can be started by an initiator and is subsequently directed at and received by a recipient. The connections between nodes can be used to calculate quantitative values that describe the shape and flow of network systems as well as the position of nodes in a network. In the current study, pigs acted as nodes and were connected via two types of edges (associations and tail biting interactions) to form two separate network types. Each pen of pigs generated an independent association network and an independent tail biting interaction network based on the behavioral data collected in each pen (as described previously). Similar to Foister et al. (18), Büttner et al. (14), and Agha et al. (19), replicates of network measures were generated by having multiple pens (*n* = 4) per litter origin; albeit sample sizes per treatment group in the present study are smaller than those in the studies cited. Additionally, repeated measures were utilized to take multiple measures from networks (measured during three observation periods). Note, data points of repeated measures were only used in the analyses of association networks given the aggregation of tail biting interaction networks. Thus, the sample size for association networks per treatment (*n* = 12 per treatment) and tail biting interaction networks per treatment (*n* = 4 per treatment) differed and produced two separate data sets. Conventional statistical analyses of social network metrics were considered adequate and permutations were not utilized given that individual pens acted as individual networks that each generated an individual data point (14, 19). Association networks were considered undirected and weighted based on HWI values (as described previously). Tail biting interaction networks were considered directed, with one pig initiating the tail biting interaction and another pig receiving the tail biting interaction. Tail biting interaction networks were weighted based on number of bites a pig received or performed regardless of the number of different pigs that a pig received bites from or that a pig bit. Each network matrix was imported into R (version 1.4.1103) (42) using the *read.xl* (version 1.3.1) package. Network matrices were transformed into edge lists and network metrics were calculated for each pen using the *igraph* (version 1.2.6) and *sna* (version 2.6) packages in R.

##### 2.3.4.1 Pen-level metrics

Pen-level (or network-level) metrics were calculated for each network type. Within association networks, three pen-level metrics that describe the shape of—and how well individuals are connected within—the network were calculated. The three metrics included: density, degree centralization, and betweenness centralization. Four pen-level metrics were calculated within tail biting interaction networks including: density, in-degree centralization, out-degree centralization, and betweenness centralization. In-degree and out-degree centralization were used due to the directionality of the tail biting interaction networks. The biological context of each pen-level metric for association networks and tail biting interaction networks is described in Table 2.

**Table 2:** Definitions of pen-level (i.e., network-level) network metrics measured within association networks and tail biting interaction networks in growing-finishing pigs.

Association network^1^	Tail biting interaction network^2^	Adapted from
**Density:** Describes the observed number of associations shared between pigs divided by the total possible number of associations between pigs (e.g., a high density value indicates all or most pigs have associated with all or most other pen-mates)	**Density:** Describes observed the number of pig dyads involved in tail biting events divided by the total number of possible pig dyads that could be involved in tail biting events (e.g., a high density value indicates all or most pigs are biting and/or are being bitten by all or most other pen-mates)	(20, 73)
**Degree centralization:** Describes the extent to which associations center around a central pig(s) in a pen (e.g., high degree centralization value indicates that a few or a single pig associate(s) with all or most other pen-mates, but other pen-mates only associate with the central pig(s)	**Out-degree centralization:** Describes extent to which a central pig(s) is/are responsible for all the biting occurring in the pen (e.g., a high out-degree centralization value indicates only a few or a single pig is biting all or most other pen-mates)	(20)
	**In-degree centralization:** Describes extent to which a central pig(s) is/are being bitten by pen-mates (e.g., a high in-degree centralization value indicates only a few or a single pig is being bitten by all or most other pen-mates)	(20)
**Betweenness centralization:** Describes the extent to which a pen possesses a few or a single pig(s) that are/is a social bridge(s) between two or more clusters of associating pigs (e.g., a high betweenness centralization value indicates a single or a few pig(s) is/are associating with only one pig in each cluster of associating pigs)	**Betweenness centralization:** Describes the extent to which a few or a single pig(s) bridges between clusters of pigs biting one another (e.g., a high betweenness centralization value indicates a single or a few pig(s) is/are biting only one pig in each cluster of biting pigs)	(20)

1 Association networks are nondirected (i.e., there is no directionality to the measurable connections between pigs, and thus there is no initiator and no recipient).

2 Tail biting interaction networks are directed (i.e., there is directionality to the measurable connections between pigs and thus there is an initiator of the interaction and a recipient).

##### 2.3.4.2 Pig-level metrics

Pig-level (or node-level) metrics were also calculated for each network type. Within association networks, two centrality metrics were calculated: weighted degree centrality (which provides information related to number and strength of paths [i.e., connections]) and betweenness centrality (which provides information related to path-lengths occurring between individuals). Degree centrality was weighted using the HWI values, meaning that degree was not based solely on how many pigs a single pig was observed lying next to, but rather based on how often the pig was observed lying with other pigs using the HWI values calculated from the raw lying data. Within the tail biting interaction networks, five centrality metrics were calculated: unweighted in-degree, unweighted out-degree, weighted in-degree centrality, weighted out-degree centrality, and betweenness centrality; however, only weighted in-degree, out-degree, and betweenness centralities were analyzed with subsequent tail biting interaction models. The biological context of each pig-level metric for the association and tail biting interaction networks is described in Table 3.

**Table 3:** Definitions of pig-level (i.e., individual-level) network metrics measured within association networks and tail biting interaction networks in growing-finishing pigs.

Association network^1^	Tail biting interaction network^2^	Adapted from
**Weighted degree centrality (i.e., overall tie strength):** Describes the combined strength of all weighted associations for an individual pig. Association interactions were weighted based on the HWI values that a pig has with other pigs over time; thus, strength represents the sum of the HWI values that an individual pig has with all other pigs in the pen (e.g., a high overall tie strength indicates a pig strongly associates with other pigs)	**Unweighted out-degree:** Describes the number of pigs that a pig has bitten. **Weighted out-degree:** Describes the combined weighted biting interactions performed by a single pig. Biting interactions were weighted based on number of bites; thus, strength represents the total number of bites a pig has performed over the observation period regardless of the number of individual pigs that the pig has bitten (e.g., a high overall tie strength indicates a pig performing a large number of bites).	(8, 68)
	**Unweighted in-degree:** Describes the number of pigs from which a pig has received bites. **Weighted in-degree:** Describes the combined weighted biting interactions received by a single pig. Biting interactions were weighted based on number of bites; thus, strength represents the total number of bites a pig has received over the observation period regardless of number of individual pigs that have bitten the pig (e.g., a high overall tie strength indicates a pig receiving a large number of bites)	(8, 68)
**Betweenness centrality:** Describes the extent to which an individual pig acts as a social bridge between clusters of pigs that prefer to associate with one another (e.g., a high betweenness centrality value indicates that a pig primarily associates with a few individuals, each from different social clusters)	**Betweenness centrality:** Describes the extent to which an individual pig acts as a behavioral bridge between clusters of pigs that bite one another (e.g., a high betweenness centrality value indicates that a pig primarily bites a few individuals, each from different clusters of biting pigs)	(68)

1 Association networks are nondirected (i.e., there is no directionality to the measurable connections between pigs, and thus there is no initiator and no recipient).

2 Tail biting interaction networks are directed (i.e., there is directionality to the measurable connections between pigs and thus there is an initiator of the interaction and a recipient).

### 2.4 Statistical analysis

Normality of all data distributions was tested using the Univariate Procedure of SAS (version 9.4; SAS Inst. Inc., Cary, NC, United States) or assessed visually using histograms in R. Growth performance data (body weight, average daily gain, average daily feed intake, and gain-to-feed ratio) were analyzed using the Mixed procedure of SAS, with litter origin, week, and the interaction as fixed effects, and residual as the random effect. Pen served as the experimental unit. Differences in time spent lying among litter origin groups were evaluated using the Glimmix procedure with Beta distribution with the same fixed effects, random effect, and experimental unit as in the Mixed procedure. Within the Mixed and Glimmix procedures, differences in means among litter origin groups were tested using the Adjusted Tukey test. Maximum tail scores were analyzed using a Kruskal Wallis Rank Sum test due to the non-normal nature of the data, with medians and their interquartile ranges (IQRs) reported. Additionally, pigs were categorized into three classes based on their MTS (MTS of 0, MTS of 1, and MTS of 2 or greater), and classes were analyzed for differences among litter origin groups using a frequency procedure with a Chi-squared test. In all cases, significant differences among means or medians were declared by *p* < 0.05 and trends by *p* < 0.10.

For analysis of social network metric data, correlation matrices were created for each data set using the *ggpairs* function of the *Ggally* (version 2.1.2) package to assess if there were any correlations among the social network metrics within each network type at each level (i.e., pig and pen-level). If the correlation coefficient among metrics was greater than 0.8, one of the metrics was removed from the analysis as suggested by Turner et al. (20). Correlation coefficients between unweighted and weighted degree centralities in tail biting interactions networks were greater than 0.8; consequently, unweighted degree metrics were excluded from the subsequent analyses.

Pen-level network metrics in both network types were not normally distributed. Thus, a univariate analysis of each SNA metric was conducted using a Kruskal Wallis Rank Sum test to evaluate litter origin effects. To assess the effect of litter origin on each individual network metric while adjusting for experimental variables present in the study (i.e., age [in instances where data were not aggregated over time] and pen), individual linear mixed models were built for each network metric. For association networks, linear mixed models using the *lmer* function in the *lme4* package (version 1.1–35.3) in R were built for each of the three metrics (density, degree centralization, and betweenness centralization) as response variables. Pen was the experimental unit and served as a random effect and litter origin and week of observation (i.e., age) were added as independent variables in the statistical models. Pairwise differences in response variables among litter origin groups were tested using the Adjusted Tukey test with any significant differences declared at a *p*-value <0.05 and trends at a *p*-value <0.10. Outcomes are reported as estimated marginal means (EMM). For tail biting interaction networks, small sample size (*n* = 12) resulting from data aggregation across observation periods prevented the construction of linear mixed models. Differences among litter origin groups were determined using a multiple comparisons test for Kruskal Wallis procedure in the *Argicolae* (version 1.3–3) package in R, with significant differences and trends determined as described for association network outcomes. Outcomes for tail biting interaction networks are reported as medians with corresponding IQR.

Pig-level network metrics were also not normally distributed. A Kruskal Wallis Rank Sum test was used to assess the effect of litter origin on response variables and then, linear mixed models were built, again to assess the effect of litter origin while adjusting for additional experimental variables present. For association network data, two models were built with one network metric serving as the response variable per model (i.e., one model for weighted degree centrality and a separate model for betweenness centrality). For each model, a pig-level network metric served as the response variable. Similarly, for tail biting interaction network data, three models were built with one network metric (i.e., weighted in-degree centrality, weighted out-degree centrality, or betweenness centrality) serving as the response variable per model. In association network models, betweenness centrality data were square root-transformed for the analysis to reduce the impact of skewness in the data distribution. Individual pig acted as the experimental unit and was nested within pen. Litter origin, week of observation (i.e., age), sex, and size of the pig acted as independent variables. Size was defined as a categorical variable based on a relative ranking of pigs’ weight within the pen close to the time of observation. Relative weight rankings within every pen included ‘small’, ‘medium’, and ‘large’, with three pigs in the pen being assigned a ‘small’ ranking, two pigs being assigned a ‘medium’ ranking, and three pigs being assigned a ‘large’ ranking. Average body weights of pigs at each age per size group across all pens are reported in Supplementary Table 1. In the aggregated data sets, size was based on the average size throughout the study period, i.e., the size category that pigs maintained most consistently across the 3 weeks of observation. Week of observation was not included in tail biting interaction network models as an independent variable because tail biting interaction networks were aggregated across observation periods. Model development used the Akaike Information Criterion (AIC) comparisons of models. Pairwise differences in the response variables among litter origin groups were determined using the same methods used for the pen-level association network data and outcomes were reported as EMM.

Given pen-level data were sparse, only pig-level data were used to test network metrics as indicators of MTS and victimization status. To determine if pig-level network metrics indicated changes in MTS, two proportional odds logistic regression models were used for analysis of the ordinal MTS data, one using association network metrics and one using tail biting interaction network metrics. The models were initially constructed with corresponding network centrality metrics (i.e., weighted degree and betweenness centralities for association networks, and weighted in-degree, weighted out-degree, and betweenness centralities for tail biting interaction networks), sex, size, and litter origin as independent variables, and MTS as the response variable. The pen in which pigs were housed was not added as a random effect due to feasibility issues within the statistical package. Betweenness centrality data were square root-transformed for the analysis to account for non-normality in the association network model. Additionally, to ensure that one network metric measurement corresponded with a MTS, each association network metric was averaged (using the mean) over the three observation periods for each pig and used for analysis. The *generalhoslem* (version 1.3.4) and *DescTools* (version 0.99.44) packages of R were used for model diagnostics. Model development used the AIC comparisons of models. Assessment of model diagnostics revealed that data did not meet assumptions of the proportional odds logistic regression model for the association network model, thus results are not reported. Based on AIC comparisons, the final tail biting interaction network model included weighted in-degree, weighted-out degree, betweenness centrality, litter origin, and sex as independent variables. For the tail biting interaction network models, results are reported as odds ratios with 95% confidence intervals with statistical significance declared at a *p*-value <0.05 and trends at a p-value <0.10.

Binomial logistic regression models were built using the *glmer* function in the *lme4* package (version 1.1–35.3) to determine if pig-level network metrics are indicative of changes in tail biting victimization status of pigs. Within the model built using association network metric data, independent variables initially included litter origin, sex, size, weighted degree centrality, and betweenness centrality. Similarly, within the model built using tail biting interaction network metric data, independent variables initially included litter origin, sex, size, weighted in-degree centrality, weighted out-degree centrality, and betweenness centrality. The corresponding pen in which each pig was housed was added as a random effect within both models. Victimization status was used as a binary (yes or no) response variable for both models. Model development, diagnostics, and fit were determined using the same methods used for the proportional odds models. Based on AIC comparisons, the final association network metric model included weighted degree, betweenness centrality, and litter origin as independent variables and the final tail biting interaction network model included weighted in-degree, weighted-out degree, betweenness centrality as independent variables. Model results are reported in a similar fashion and using the same significance cut off values.

## 3 Results

### 3.1 Effect of litter origin on growth performance, maximal tail injury score, and time budget for lying posture

There were no differences in body weight at the beginning nor at the end of the study period among litter origin groups (Table 4). Litter origin had no effect (all *p* > 0.12) on average daily gain, average daily feed intake, or gain-to-feed ratio. No interaction of litter origin with the week of the study period on growth performance was detected.

**Table 4:** Effect of litter origin on growth performance and maximal tail injury scores^1^ of growing-finishing pigs.

Item	Litter origin^2^			SE	*p-*value
	Littermates	Half-littermates	Non-littermates		
# of pens	4	4	4		
Pigs/pen	8	8	8		
Body weight, kg					
Initial weight^3^	24.5	25.7	25.7	0.52	0.24
Final weight^4^	121.4	124.1	124.7	1.26	0.19
ADG^5^^,^^6^, g	984	985	1,005	16.72	0.62
ADFI^5^^,^^7^, g	2,613	2,613	2,575	40	0.74
Gain:Feed	0.40	0.40	0.41	0.01	0.12
Maximum Tail Score^8^					
Median (IQR)^9^	1.5 (1.0–2.0)^a^	1 (1.0–2.0)^ab^	1 (0–1.0)^b^	–	0.009
Pigs with Score 0^10^	1 (3.1%)	3 (9.4%)	6 (18.8%)	11.6^11^	0.02
Pigs with Score 1^10^	12 (37.5%)	18 (56.3%)	19 (59.4%)		
Pigs with Score ≥ 2^10^	19 (59.4%)	11 (34.4%)	7 (21.9%)		

1 Tail injury was scored using a subjective system (ranging from 0 to 4) described by Kritas and Morrison (35) and Li et al. (8).

2 Littermate pens consisted of pigs that were farrowed and nursed by the same sow; Half-littermate pens consisted of two sets of four pigs farrowed and nursed by one sow per set; Non-littermate pens consisted of eight pigs that were each farrowed and nursed by a different sow.

3 10 weeks of age.

4 24 weeks of age.

5 10–24 weeks.

6 Average Daily Gain.

7 Average Daily Feed Intake.

8 The maximum injury injury score for each pig over the 14 weeks of the study period, assessed twice per week and during tail biting outbreaks. Tail score 0 = no observable injury; tail score 1 = Healed lesion with minor scabs; tail score 2 = visible blood on the tail without sign of infection; tail score 3 = open wound with signs of infection; tail score 4 = partial loss of the tail with signs of infection.

9 Interquartile range (25 to 75%); Medians within a row without a common superscript differ.

10 Number of pigs with the maximum tail score 0, 1, 2 or greater; (Percentage of pigs [*n* = 32] assigned to each treatment group).

11 Chi-square value (df = 4).

Litter origin affected tail injury as measured by the MTS (Table 4). Pigs in LM pens had a higher median MTS compared to pigs in NLM pens. Pigs in HLM pens had no difference in medians of MTS when compared to pigs in LM and NLM pens (*p* = 0.009). When assessing distribution of MTS across litter origin treatments, LM pens had the highest percentage of pigs victimized by tail biting, with MTS of 2 or greater compared to HLM and NLM pens, and the lowest percent of pigs with MTS of 0 compared to HLM and NLM pens (*p* = 0.02). The time that pigs spent lying was not influenced by litter origin (*p* = 0.54) or week of the study period (*p* = 0.41). There was a tendency (*p* = 0.096) of interaction between litter origin and week, so lying behavior data were examined for each week. No litter origin effect was detected at any observation timepoint during the study (*p* > 0.10; Supplementary Table S2).

### 3.2 Effect of litter origin on association network metrics

#### 3.2.1 Pen level

No interactions between litter origin and week of observation (i.e., age of pigs) on association network metrics at the pen-level were detected, except for degree centralization. The main effects of litter origin did not influence density (Table 5), indicating that pens had similar ratios of connections regardless of litter origin treatment. Medians of density across litter origin treatments ranged between 0.81 and 0.87, suggesting that all association networks were dense. Litter origin had no effect on betweenness centralization. Medians of betweenness centralization were 0.04 or lower across litter origin, indicating that the network shape exhibited a limited presence of distinct clusters of associating pigs bridged by individuals. The age of pigs also had no effect on density or betweenness centralization (Table 6).

**Table 5:** Effect of litter origin on pen-level association network metrics^1^ for growing-finishing pigs^2^.

Social network metric	Litter origin^3^			Pooled SE	*p*-value
	Littermates	Half-littermates	Non-littermates		
Density	0.81 (0.76–0.86)	0.81 (0.76–0.86)	0.87 (0.82–0.92)	0.02	0.15
Betweenness Centralization	0.04 (0.02–0.06)	0.04 (0.02–0.05)	0.02 (0.002–0.03)	0.01	0.13

1 Association networks were built using half-weight association index (HWI). HWI = x/(n_a_ + n_b_)/2; x = the number of times pig a and pig b are lying together during 6 h of the observation period per week for 3 weeks; n_a_ is the number of times pig a is lying during the same period; n_b_ is the number of times pig b is lying during the same period. Values in the table are reported as Estimated Marginal Means (EMM) with lower and upper limits for 95% Confidence Intervals in the parentheses.

2 Results of multiple linear mixed models with a social network metric serving as the response variable in each model.

3 Littermate pens consisted of pigs that were farrowed and nursed by the same sow; Half-littermate pens consisted of two sets of four pigs farrowed and nursed by one sow per set; Non-littermate pens consisted of eight pigs that were each farrowed and nursed by a different sow. There were four pens per litter origin treatment.

**Table 6:** Effect of age on pen-level association network^1^ metrics for growing-finishing pigs^2^.

Social network metric	Age			Pooled SE	*p*-value
	15 weeks	19 weeks	23 weeks		
Density	0.86 (0.82–0.91)	0.81 (0.76–0.85)	0.82 (0.77–0.87)	0.02	0.20
Betweenness Centralization	0.03 (0.01–0.04)	0.03 (0.02–0.05)	0.04 (0.02–0.05)	0.01	0.49

1 Association networks were built using half-weight association index (HWI). HWI = x/(n_a_ + n_b_)/2; x = the number of times pig a and pig b are lying together during 6 h of the observation period; n_a_ is the number of times pig a is lying during the same period; n_b_ is the number of times pig b is lying during the same period. Values in the table are reported as Estimated Marginal Means (EMM) with lower and upper limits for 95% Confidence Intervals in the parentheses.

2 Results of multiple linear mixed models with a social network metric serving as the response variable in each model.

The effect of litter origin on degree centralization was dependent upon the age of pigs (Table 7). Non-littermate pens had lower degree centralization compared to LM and HLM pens at both 15 weeks (*p* = 0.003) and 23 weeks (*p* = 0.01). At 19 weeks of age, litter origin did not affect degree centralization. In other words, the association networks that formed in NLM pens had less of a spoke-and-wheel network shape that centered a well-connected pig whose contacts had few connections with other pigs than LM and HLM pens.

**Table 7:** Effect of litter origin on degree centralization in association networks^1^ for growing-finishing pigs at different ages.

Age	Litter origin^2^			SE	*p-*value
	Littermates	Half-littermates	Non-littermates		
15 weeks^3^	0.14 (0.09–0.19)^a^	0.20 (0.15–0.25)^a^	0.07 (0.02–0.12)^b^	0.03	0.003
19 weeks	0.13 (0.07–0.18)	0.13 (0.07–0.18)	0.15 (0.10–0.20)	0.03	0.74
23 weeks^3^	0.21 (0.15–0.26)^a^	0.14 (0.09–0.19)^a^	0.09 (0.04–0.14)^b^	0.03	0.01

1 Association networks were built using half-weight association index (HWI). HWI = x/(n_a_ + n_b_)/2; x = the number of times pig a and pig b are lying together during 6 h of the observation period; n_a_ is the number of times pig a is lying during the same period; n_b_ is the number of times pig b is lying during the same period. Values in the table are reported as Estimated Marginal Means (EMM) with lower and upper limits for 95% Confidence Intervals in the parentheses.

2 Littermate pens consisted of pigs that were farrowed and nursed by the same sow; Half-littermate pens consisted of two sets of four pigs farrowed and nursed by one sow per set; Non-littermate pens consisted of eight pigs that were each farrowed and nursed by a different sow. There were four pens per litter origin treatment.

3 EMMs without a common superscript differ (*p* < 0.05).

#### 3.2.2 Pig level

Litter origin affected weighted degree centrality of individual pigs, with pigs in NLM pens having higher weighted degree centrality compared to pigs in LM and HLM pens (*p* = 0.002; Table 8). This result suggests that pigs in NLM pens had a higher number and strength of associations with their pen-mates compared to pigs in LM and HLM pens. In contrast, litter origin had no effect on betweenness centrality of individual pigs, meaning there was no difference in pigs’ propensity to act as bridges between social clusters in association networks across litter origin treatments. Sex, size, and week of observation did not affect either weighted degree centrality or betweenness centrality.

**Table 8:** Effect of litter origin on pig-level association network^1^ metrics for growing-finishing pigs^2^.

Social network metric	Litter origin^3^			Pooled SE	*p*-value
	Littermates	Half-littermates	Non-littermates		
Weighted Degree Centrality^4^	0.76 (0.66–0.86)^a^	0.86 (0.76–0.96)^a^	1.00 (0.90–1.11)^b^	0.05	0.002
Betweenness Centrality	1.31 (1.07–1.56)	1.18 (0.93–1.43)	1.26 (1.02–1.51)	0.11	0.68

1 Association networks were built using half-weight association index (HWI). HWI = x/(n_a_ + n_b_)/2; x = the number of times pig a and pig b are lying together during 6 h of the observation period per week for 3 weeks; n_a_ is the number of times pig a is lying during the same period; n_b_ is the number of times pig b is lying during the same period. Values in the table are reported as Estimated Marginal Means (EMM) with lower and upper limits for 95% Confidence Intervals in the parentheses.

2 Results of multiple linear mixed models with a social network metric serving as the response variable in each model.

3 Littermate pens consisted of pigs that were farrowed and nursed by the same sow; Half-littermate pens consisted of two sets of four pigs farrowed and nursed by one sow per set; Non-littermate pens consisted of eight pigs that were each farrowed and nursed by a different sow. There were four pens per litter origin treatment.

4 EMMs without a common superscript differ (*p* < 0.05).

### 3.3 Effect of litter origin on tail biting network metrics

#### 3.3.1 Pen level

Litter origin had no impact on any pen-level metrics including density, out-degree centralization, in-degree centralization, and betweenness centralization of tail biting networks (Table 9). Medians of density across litter origin groups ranged between 0.12 and 0.22, suggesting that all tail biting networks were sparse regardless of litter origin group. Out-degree centralization medians across litter origin groups were closer to values of 0 than 1.0 (i.e., between 0.24 and 0.34), demonstrating that tail biting networks generally lacked an outwardly directed spoke-and-wheel network shape and no pig acted as the only perpetrator of tail biting toward numerous pen-mates. In-degree centralization medians across litter origin groups were less than 0.2 (ranging between 0.17 and 0.19). In-degree centralization medians that are closer to 0 than 1.0 suggest that tail biting networks across all treatment groups lacked an inwardly directed spoke-and-wheel network shape and that no specific pigs tended to be singled out by other pigs in the pen as a target for tail biting within pens. Likewise, medians of betweenness centralization were less than 0.2 across litter origin groups (ranging between 0.08 and 0.13), indicating that individual pigs did not bridge any of tail biting interaction clusters (i.e., groups of pigs that were biting each other) across all treatment groups.

**Table 9:** Effect of litter origin on pen-level tail biting interaction network^1^ metrics for growing-finishing pigs^2^.

Social network metric	Litter origin^3^			Chi-square value	df	*p-*value
	Littermates	Half-littermates	Non-littermates			
Density	0.17 (0.13–0.18)	0.22 (0.17–0.26)	0.12 (0.07–0.17)	3.16	2	0.21
Out-degree Centralization	0.30 (0.27–0.33)	0.34 (0.30–0.45)	0.24 (0.23–0.25)	3.90	2	0.14
In-degree Centralization	0.19 (0.16–0.20)	0.17 (0.13–0.20)	0.17 (0.08–0.25)	0.48	2	0.79
Betweenness Centralization	0.11 (0.06–0.14)	0.13 (0.05–0.18)	0.08 (0.02–0.09)	1.67	2	0.44

1 Tail biting networks were built based on the number of tail biting interactions among pigs in each pen during 6 h of the observation period per week for 3 weeks.

2 Results of multiple Kruskal Wallis Rank Sum tests with a social network metric serving as the response variable in each test.

3 Littermate pens consisted of pigs that were farrowed and nursed by the same sow; Half-littermate pens consisted of two sets of four pigs farrowed and nursed by one sow per set; Non-littermate pens consisted of eight pigs that were each farrowed and nursed by a different sow. There were four pens per litter origin treatment. Values in the table are reported as reported as median with interquartile range (25 to 75%) in the parentheses.

#### 3.3.2 Pig level

Litter origin had no effect on weighted out-degree centrality or betweenness centrality of individual pigs (both *p* > 0.11; Table 10). Such findings indicate that the number of tail bites performed by individual pigs and the capacity of an individual pig to be a bridge between clusters of tail biting interactions were not impacted by litter origin treatment. However, weighted in-degree centrality tended to be higher in HLM pigs compared to pigs in LM and NLM pens (*p* = 0.09), meaning some pigs in HLM pens tended to receive more bites from their pen-mates compared to pigs in other treatment pens. Sex and average size did not affect weighted in-degree, weighted out-degree, or betweenness centralities.

**Table 10:** Effect of litter origin on pig-level tail biting interaction network metrics^1^ for growing-finishing pigs^2^.

Social network metric	Litter origin^3^			Pooled-SE	*p*-value
	Littermates	Half-littermates	Non-littermates		
Weighted Out-Degree Centrality (strength)	1.10 (0.22–1.98)	1.64 (0.59–2.69)	0.55 (−0.50–1.60)	0.50	0.11
Weighted In-Degree Centrality (strength)^4^	1.28 (0.48–2.08)^a^^,^^b^	2.27 (1.38–3.16)^b^	1.15 (0.26–2.04)^a^	0.44	0.09
Betweenness Centrality	3.75 (−0.61–8.11)	1.40 (−3.12–5.92)	−0.17 (−4.68–4.35)	2.18	0.38

1 Tail biting interaction networks were built based on the number of tail biting interactions among pigs in each pen during 6 h of the observation period per week for 3 weeks.

2 Results of multiple linear mixed models with a social network metric serving as the response variable in each model.

3 Littermate pens consisted of pigs that were farrowed and nursed by the same sow; Half-littermate pens consisted of two sets of four pigs farrowed and nursed by one sow per set; Non-littermate pens consisted of eight pigs that were each farrowed and nursed by a different sow. There were four pens per litter origin treatment. Values in the table are reported as Estimated Marginal Means (EMM) with lower and upper limits for 95% Confidence Intervals (CI) in the parentheses.

4 Means within a row without a common superscript tend to differ (*p* < 0.10).

### 3.4 Indicators of maximal tail injury score

#### 3.4.1 Tail biting network metrics as indicators of maximal tail injury score

Weighted in-degree centrality in tail biting networks was indicative of changes in MTS when holding all other variables constant. Every one unit increase in weighted in-degree centrality increased the odds of receiving a more severe MTS by 1.56 times (95% CI = 1.08–2.27; *p* = 0.02; Table 11). These findings suggest a positive relationship between weighted in-degree centrality (i.e., number and strength of contact) and the risk of a pig receiving a more severe MTS. Additionally, every one unit increase in weighted out-degree centrality tended to increase the odds of an increase in MTS by 1.19 times (95% CI = 0.97–1.48; *p* = 0.09; Table 11). Changes in betweenness centrality were not associated with any change in odds that a pig would have a more severe MTS.

**Table 11:** Odds ratios of tail biting interaction network^1^ centrality metrics as indicators of increased maximal tail injury score^2^.

Centrality metric as indicator	Estimate	Odds ratio (95% CI)^3^	*p-*value
Weighted Out-degree Centrality	0.18	1.19 (0.97–1.48)	0.09
Weighted In-degree Centrality	0.44	1.56 (1.08–2.27)	0.02
Betweenness Centrality	−0.03	0.97 (0.88–1.07)	0.58

1 Tail biting interaction networks were built based on the number of tail biting interactions among pigs in each pen during 6 h of the observation period per week for three weeks.

2 Tail injury was scored using a subjective system (ranging from 0 to 4) described by Kritas and Morrison (35) and Li et al. (8).

3 Lower and upper limits for 95% Confidence Intervals in the parentheses.

#### 3.4.2 Other variables as indicators of maximal tail injury score

Litter origin was indicative of receiving increased MTS (*p* = 0.02). Being in a LM pen increased the odds that a pig would receive an increased MTS compared to being in a NLM pen by 4.27 times (95% CI = 1.57–12.23; *p* = 0.005) (data not shown in the table). Sex was not indicative of pigs receiving an increased MTS (*p* > 0.18). Changes in model fit statistics when specific independent variables were removed from the null model are presented in Supplementary Table S3.

### 3.5 Indicators of pigs becoming victims of tail biting

#### 3.5.1 Association network metrics as indicators of pigs becoming victims of tail biting

No association network metrics were indicative of pigs becoming victims of tail biting (Table 12). Such findings indicate that neither the number and strength of associations between pigs nor the capacity to which an individual pig may be a bridge between clusters of associating pigs indicate if a pig would become a victim of tail biting within a pen. Litter origin was not an indicator for pigs becoming victims of tail biting at any point in the study.

**Table 12:** Odds ratios of association network^1^ centrality metrics as indicators of pigs becoming victims of tail biting^2^.

Centrality metric as indicator	Estimate	Odds ratio (95% CI)^3^	*p-*value
Weighted Degree Centrality	0.71	2.03 (0.02–195.44)	0.77
Betweenness Centrality	0.06	1.06 (0.32–3.80)	0.93

1 Association networks were built using half-weight association index (HWI). HWI = x/(n_a_ + n_b_)/2; x = the number of times pig a and pig b are lying together during 6 h of the observation period per week for 3 weeks; n_a_ is the number of times pig a is lying during the same period; n_b_ is the number of times pig b is lying during the same period.

2 Tail injury was scored using a subjective system (ranging from 0 to 4) described by Kritas and Morrison (35) and Li et al. (8). Pigs that received a score of 2 or greater were considered victims of tail biting.

3 Lower and upper limits for 95% Confidence Intervals in the parentheses.

#### 3.5.2 Tail biting network metrics as indicators of pigs becoming victims of tail biting

No tail biting interaction network metrics were indicative of pigs becoming victims of tail biting (Table 13). Such findings indicate that the number of tail bites performed or received by individual pigs as well as the capacity to which an individual pig may be a bridge between clusters of tail biting interactions do not indicate if a pig would become a victim of tail biting within a pen.

**Table 13:** Odds ratios of tail biting interaction network^1^ centrality metrics as indicators of pigs becoming victims of tail biting^2^.

Centrality metric as indicator	Estimate	Odds ratio (95% CI)^3^	*p-*value
Weighted Out-degree Centrality	0.10	1.10 (0.80–1.51)	0.54
Weighted In-degree Centrality	0.18	1.20 (0.70–2.03)	0.49
Betweenness Centrality	0.02	1.02 (0.86–1.20)	0.83

1 Tail biting interaction networks were built based on the number of tail biting interactions among pigs in each pen during 6 h of the observation period per week for 3 weeks.

2 Tail injury was scored using a subjective system (ranging from 0 to 4) described by Kritas and Morrison (35) and Li et al. (8). Pigs that received a score of 2 or greater were considered victims of tail biting.

3 Lower and upper limits for 95% Confidence Intervals in the parentheses.

## 4 Discussion

The goal of the present study was to assess whether tail injury caused by tail biting behavior could be predicted using SNA, specifically analyses of pig association networks and tail biting interaction networks. The impact of litter origin on social network metrics was evaluated as a means to understand the dynamics of social structures in pigs and, subsequently, the pig-level and pen-level social network metrics were evaluated as potential indicators of tail damage. The effect of litter origin on growth measures was also assessed.

Litter origin did not have an impact on growth performance of growing-finishing pigs. Pigs in LM pens had a higher median MTS and higher proportions of pigs victimized by tail biting compared to other litter origin groups. Analysis of association network metrics indicate that litter origin only affected degree centralization—specifically at 15 and 23 weeks of age. Additionally, NLM pens exhibited lower degree centralization compared to LM and HLM pens, but no differences in density were observed between litter origin groups. At a pig-level, pigs in NLM pens had higher weighted degree centrality in their association networks compared to pigs in HLM and LM pens. When using network metrics to indicate if pigs would become victims of tail biting, none of the association network metrics and none of the tail biting interaction network metrics were indicative of any changes in the risk that a pig would become a victim of tail biting. However, changes in the weighted in-degree centrality within a pig’s tail biting interaction network, independent of litter origin group, were indicative of changes in MTS, with high weighted in-degree centrality being associated with increased risk of incurring a more severe MTS. Pigs with higher weighted out-degree centrality tended to be at a higher risk for receiving a more severe MTS, suggesting that tail biters tended to be more at risk of severe tail injury. Finally, pigs in NLM groups did not have lower weighted in-or out-degree centralities within the tail biting interaction networks despite having lower median MTS and the smallest proportion of pigs victimized by tail biting compared to LM groups.

Such findings related to litter origin not affecting growth performance of growing finishing pigs are consistent with results of one previous work that investigated the effects of litter origin on nursery pigs (8), albeit researchers used the same herd and genetics as used in the present study. Other researchers demonstrated that litter origin affects weight gain in nursery pigs (10) and growing-finishing pigs (43). In such studies (10, 43), littermate groups obviously were not mixed with unfamiliar pigs, but non-littermate groups were mixed with unfamiliar pigs prior to the study in order to create the treatment groups. The effect of mixing unfamiliar pigs and related stress that occurs when pigs are introduced to individuals who they are not familiar with may have confounded the effect of litter origin within these studies. Stookey and Gonyou (44) noted that in groups of growing-finishing pigs that were recently mixed with unfamiliar pigs, mixed groups had lower weight gain than groups of unmixed pigs; however, the weight loss was only observed within 2 weeks of mixing (44). Thus, it is not surprising that we did not detect the effect of litter origin on growth performance of pigs in the present study because our pigs were mixed 6 weeks before the start of data collection.

The results of LM pigs having a higher median MTS and higher proportions of pigs that became victims of tail biting mirrors previous findings in nursery pigs (8). However, a study by Veit et al. (45) found no link between retaining groups of littermates post-weaning and tail injury score in undocked nursery pigs (45). Veit et al. (45) recorded the frequencies of tail injury scores during 2 through 6 weeks post-weaning in contrast to the 14 weeks of monitoring the growing-finishing pigs in the present study. Veit et al. (45) also explored differences in loss of tail—which more closely resembles a MTS of 4 in the current study—between “mixed” litter (i.e., pigs from three separate litters were mixed) and littermate groups, and did not detect differences between the groups. The discrepancy between the current study and the study reported by Veit et al. (45) could be attributed to the different methods used to assess tail injury. Our results suggest that pigs in LM groups endure more tail injuries as indicated by higher MTS and by the observation that pigs in LM groups are more often victimized by tail biting compared to pigs in other litter origin groups. Here, we attempt to explain the difference in MTS among litter origin groups through analysis of the social network metrics.

The results of pigs in NLM pens having lower degree centralization in association networks indicate that NLM pens broadly had a more even distribution of weighted degree centrality across pen-mates and that NLM pens were less centralized. Analysis of association networks at the pig-level indicated that pigs in NLM pens generally had higher weighted degree centrality compared to pigs in LM and HLM pens. Metrics from association networks suggest that across pen-mates within NLM pens, pigs had more uniform numbers of affiliative connections with other pen-mates and more numerous and stronger affiliative connections, as indicated by resting in bodily contact, compared to pigs in LM and HLM pens. Li et al. (5) observed similar findings in pig-level metrics from association networks formed by nursery pigs after weaning in the same herd and genetics used in the present study. Other researchers that assessed affiliative dyad formation in pigs found that relatedness was not a strong driver of affiliation in either nursery pigs (6, 7) or growing-finishing pigs (46). We speculate that the present findings may be more related to the development (or lack of development) of pigs’ social skills and the contributions of such skills to long term stability of social groups rather than their relatedness. There is evidence that introduction of pigs to non-littermates pre-weaning allows pigs to develop social skills that can aid in preserving stability within social groups after weaning (23, 47–51). In the present study, uniformity, strength, and number of affiliations observed in association networks of NLM pens, as indicated by low degree centralization in pens and high weighted-degree centrality of pigs, could be related to the social skills that pigs in NLM pens had to learn during mixing at weaning. This observation is supported by the differences in such metrics observed in pigs in LM pens, which did not experience interactions with non-littermates throughout their life; however, findings being due to social skill development is still a very speculative idea. Further research is needed to separate impact of socialization with unfamiliar pigs through mixing at weaning from the impact of relatedness on social structures of pigs to better understand if findings are truly related to social skill development.

Interestingly, while Li et al. (8) observed differences in association network densities between pens of different litter origin groups in nursery age pigs, litter origin treatment groups had no effect on the density of the finishing pigs’ association networks in the present study. However, the lack of difference in association network density between litter origin groups and the general trend of high-density networks across all pens in the present study was not entirely unexpected given the low space allowance per pig as well as given the limited number of pen-mates within the pens (i.e., the small network size). Future assessment of density using networks formed by strong ties (i.e., ties with a specific minimum value cut off point) would likely yield additional insights, given that strong ties as described in Durrell et al. (7) and Li et al. (8) are suggestive of more stable associative relationships between pigs.

When observing that pigs in NLM pens had low median MTS in tandem with the trends observed in association networks, results suggest that the low MTS incurred by NLM pigs may be related to pigs being in a social setting where individuals are uniformly and well affiliated with one another. The existence of a relationship between tail damage and association network metrics was also observed by Li et al. (8) in nursery pigs. Increased incidence of affiliative behavior, as measured by behavior of pigs lying together, may be a means for pigs to cope with anxiety that stems from being the recipient of agonistic behavior (33). One might speculate that pigs in NLM pens developed stronger social skills compared to pigs in LM pens in order to cope with potential social stress.

Social skills potentially could contribute to the dynamics of affiliative behavior as observed in their association networks and could improve the ability of pigs in NLM groups to more readily receive and provide social support from and to pen-mates. Researchers have observed that social support acts as a mechanism that animals (52), including pigs, use to cope with stressors, such as stress related to being involvement with aggressive behaviors (33, 53), exposure to restraint (54), isolation (54), white noise (55), and immersion in foam (56). Possibly, social support among pigs could help mediate stresses in swine production by reducing the presentation of maladaptive coping strategies such as tail biting behavior (57). Klein et al. (58) suggested that social skills learned during pre-weaning socialization with non-littermates can improve maintenance of intact tails post-weaning and into the fattening period. However, given that no agonistic behaviors outside of tail biting were measured in the present study, NLM pens could possibly experience higher incidence of these behaviors and perhaps experience a greater need for the use of social support that went undetected within the present study. Additional, research examining how and when social support is used in groups of growing-finishing pigs in different litter origin-based groups is needed.

Results surrounding weighted in-degree and weighted out-degree centrality being associated with an increased risk of having a more severe MTS are in line in some capacity with findings of Ursinus et al. (59). In their study, Ursinus et al. (59) reported correlations between tail damage and tail biting behavior, observing that tail damage at the growing phase was associated with tail biting behavior at the finishing phase of pigs, suggesting that biters may be predisposed to tail damage. However, it should be noted that because there was not a feasible way to add the random effect of the pen in which pigs were housed into the proportional odds model used in this study, the independence of data is not completely accounted for; thus, this is an important limitation when interpreting the effects observed within these results. Although, while pen was not accounted for in the proportional odds model, weighted in-degree and weighted out degree in the tail biting interaction network binomial logit regression model had effects in the same direction as those observed in the proportional odds model. Such findings observed in tandem can somewhat validate the impact these social network metrics may have on a pig’s risk of an increasing MTS but only to a very limited extent given that no significance was observed in the logit models.

There are other limitations to this study that need to be considered before extrapolating findings to commercial swine production systems. Specifically, while social networks as represented by multiple individual pens and repeated measures over time allowed for use of conventional statistics to analyze pen-and pig-level measures without the need to permute networks, the sample size at the pen-level was very small. Such a small sample size is a severe limitation and provides an incredibly diminished statistical power when using conventional statistics to explore the effect of variables on social network metrics or the effect of social network metrics on other variables. The sample size in the present study was a result of space and time restrictions within the research facility. Findings of the present study still provide important preliminary insights, but future studies need to utilize increased sample sizes to provide additional value.

Further, the behavioral observation data were collected and recorded manually. The labor and time required to record the data was intensive which was a huge limitation for this study and other studies of tail biting in pigs (60). Behavioral data collection could be improved vastly by utilizing new technology such as automation and machine learning (61). Future studies of tail biting through SNA could also benefit from continuously recording behavior for extended periods to build more thoroughly populated network matrices.

In addition, the behavioral observation period (6 h every 3 weeks: 18 h total) used to build tail biting interaction networks in this study was very limited in comparison to the entire growing-finishing period (14 weeks: 2,352 h total). Thus, a large number of tail biting interactions likely occurred outside the observation period, and not all the interactions contributing to tail damage were recorded in the network data set.

Further, the pen size used in the current study was smaller than most pen sizes used in commercial production (62, 63). The small pen size limits a pig’s options as to where they can lie down. Thus, it is possible that association networks based on lying behavior—in such small spaces—could be biased by motivations unrelated to social preferences such as, e.g., maintaining thermoregulation (64) or location preferences (65). Moreover, networks with eight pigs are considered small networks (66), and network-level characteristics, such as network shape and network flow-through, are not as likely to be detected in comparison to larger, more populated networks. As of 2024, there are few established analytical tools presently available for the comparison of multiple small networks (17). Most previous researchers examined behavior and spatial proximity in a single large population of animals, including captive animals in a zoo (67), wildlife populations in field environments (68), and livestock (69). But there are novel types of analyses that aim to use multiple small networks as replicates that are being explored (66). Thus researchers in the field of animal behavior are beginning to examine ways in which data from multiple small networks can be analyzed to yield meaningful findings (8, 70).

Moreover, given that researchers have demonstrated that group size does impact social behavior of pigs, network dynamics with the present study likely do not reflect the same network dynamics present in networks measured from larger pen sizes used in commercial growing-finishing pig facilities in major pig-producing countries (62, 63). Thus, further studies are needed to yield findings that are directly applicable to commercial settings.

Finally, there are three types of tail biting behavior which include: two-staged, sudden forceful, and obsessive (29). In the current study, the tail biting behavior data were primarily based on two; sudden-forceful and obsessive tail biting. These types of biting are characterized by the recipient of a bite having a noticeable reaction when bitten. The two-staged tail biting behavior, in which recipients of a bite are characteristically passive and/or apathetic, was likely not captured during data collection in the present study. It is possible that two-staged tail biting may have been occurring and acting as a substantial contributor to tail damage in the current study, but was not accounted for in our analyses. Other researchers note that two-stage tail biting does readily contribute to tail damage. Zonderland et al. (71) monitored tail biting behavior in nursery pigs over 7 days and concluded that two-staged tail biting was the predominant type of tail biting occurring and causing the most tail damage compared to sudden forceful or obsessive tail biting. Bagaria et al. (72) also observed two-staged tail biting as the predominant type of tail biting in 8-week-old pigs. Thus, further research is needed to assess the contributions of each type of tail biting behavior to tail damage.

## 5 Conclusion

In this study the capacity to which tail injury caused by tail biting behavior could be indicated using social network metrics was explored. Additionally, the impact of litter origin on social network metrics from association and tail biting interaction networks were evaluated as a means to understand the dynamics of social structures in pigs, and the potential for using pig-level and pen-level network metrics on indicators of tail damage was assessed. Results of the current study suggest that litter origin influences MTS, with pigs in LM pens possessing the highest MTS and the largest proportion of pigs with tail injury scores that qualified pigs as victims of tail biting. Additionally, pigs from NLM pens appeared to exhibit the strongest and most uniform social connectedness and had the lowest MTS among litter origin groups. Weighted in-degree centrality and, to a lesser extent, weighted out-degree centrality within tail biting interaction networks could potentially act as indicators of increased MTS. Findings of this exploratory study imply that social structures of pigs can impact tail biting behavior and tail damage caused by tail biting. Future studies are needed to build on the current findings by collecting behavioral data continuously for extended periods, by using larger network sample sizes, and by verifying the findings in larger group sizes that are more representative of commercial production settings.

## Data Availability

The raw data supporting the conclusions of this article will be made available by the authors, without undue reservation.
