# Supplementary material for: Evaluating social network metrics as indicators of tail injury caused by tail biting in growing-finishing pigs (Sus scrofa domesticus)
Source: Front Vet Sci. 2024 Sep 27;11:1441813. doi: 10.3389/fvets.2024.1441813 (PMC11466945; doi:10.3389/fvets.2024.1441813)
Supplement: Supplementary file 1 [file Table_1.docx]

Supplementary Material

**Table S1. Mean body weight (kg) of pigs across all pens by size group over time (i.e., at each week of observation).**

| Age^1^ | Large | Medium | Small |
| --- | --- | --- | --- |
| 15 | 129.5 | 120.8 | 112.8 |
| 19 | 195.0 | 182.9 | 171.8 |
| 23 | 260.6 | 247.5 | 233.1 |
| ^1^Weeks of age | | | |

**Table S2. Effect of litter origin on time budget for lying posture of growing-finishing pigs**

| Item | Litter Origin^1^ | | | SE | *p-*value | |
| --- | --- | --- | --- | --- | --- | --- |
|  | Littermates | Half-Littermates | Non-Littermates |  | | |
| Time budget for lying posture, % of observation time^2^ | | | | | |  |
| 15 weeks of age | 79.0 | 79.9 | 81.1 | 1.35 | | 0.56 |
| 19 weeks of age | 80.7 | 80.4 | 77.1 | 1.55 | | 0.23 |
| 23 weeks of age | 80.8 | 75.5 | 78.6 | 1.57 | | 0.10 |
| ^1^Littermate pens consisted of pigs that were farrowed and nursed by the same sow; Half-littermate pens consisted of two sets of four pigs farrowed and nursed by one sow per set; Non-littermate pens consisted of eight pigs that were each farrowed and nursed by a different sow.  ^2^Six hours per pen each week. There were four pens per litter origin treatment. | | | | | | |

**Table S3. Proportional odds model fit statistics when compared with the null hypothesis model^1^ assessing tail biting interaction network^2^ centrality metrics as indicators of increased maximal tail injury score^3^**

| Independent Variable | Change in Model Fit Statistic | | |
| --- | --- | --- | --- |
|  | χ2 | df | *p*-value |
| Weighted Out-degree Centrality | 2.87 | 1 | 0.09 |
| Weighted In-degree Centrality | 5.54 | 1 | 0.02 |
| Betweenness Centrality | 0.30 | 1 | 0.58 |
| Litter Origin | 8.82 | 2 | 0.01 |
| Sex | 1.78 | 1 | 0.18 |
| ^1^Full model: Response variable for null model: Tail injury score; Independent variables: Weighted in-degree centrality + Weighted out-degree centrality + Betweenness centrality + Litter Origin Treatment + Sex + Size.  ^2^Tail biting interaction networks were built based on the number of tail biting interactions between pigs in each pen during 6 hours on each observation day.  ^3^Tail injury was scored using a subjective system (ranging from 0 to 4) described by Kritas and Morrison (1) and Li et al. (2). | | | |

References for Table S3:

1. Kritas SK, Morrison RB. An observational study on tail biting in commercial grower-finisher barns. J Swine Health Product. (2004) 12:17–22. doi: 10.54846/jshap/374

2. Li Y, Zhang H, Johnston LJ, Martin W. Understanding tail-biting in pigs through social network analysis. Animals. (2018) 8(1), 13. doi: 10.3390/ani8010013
